# RETRACTION: Chelerythrine Inhibits Stemness of Cancer Stem-Like Cells of Osteosarcoma and PI3K/AKT/mTOR Signal

**DOI:** 10.1155/jo/9832873

**Published:** 2025-11-14

**Authors:** Journal of Oncology

**RETRACTION**: Z. Chen, H. Yang, Q. Zhang, Q. Hu, and Z. Zhao, “Chelerythrine Inhibits Stemness of Cancer Stem-Like Cells of Osteosarcoma and PI3K/AKT/mTOR Signal,” *Journal of Oncology,* no. 2022 (2022), https://doi.org/10.1155/2022/6435431.

The above article, published online on 12 September 2022 in Wiley Online Library (https://www.wileyonlinelibrary.com), has been retracted by John Wiley & Sons Ltd.

The retraction has been agreed following an investigation of the concerns flagged by *Mycosphaerella arachidis* and *Hoya Camphorifolia* on PubPeer [1], which identified multiple instances of figure duplication.

Specifically:

- Figure 2c: The cancer stem-like cells shown in the 2 panels on the left appear similar to the two images of HCT116 cells on the left side of Figure 2c of [2].
- Figure 5a: The WB bands corresponding to the expression of MMP-2 and MMP-9 are identical to the bands from Figure 3c of [3], where they represent the expression of different biomarkers in a different cell type.
- Figure 6a: The bands corresponding to the expression of p-PI3K in U2OS cells and P-AKT in MG-63 cells share unexpected similarities. Additional concerns were also identified related to the similarity of the bands showing the expression of PI3K in U2OS cells to the CHE-Nanog bands in Figure 3a.
- Figure 7: The image of the mouse on the right-hand side of panel “a” is unexpectedly similar to Figure 7 of [4], where the mouse was used in a different experiment. Moreover, the CHE tumors shown in panel “c” also appear identical to the shCCDC88A tumors shown in Figure 6c of the abovementioned paper.

As a result of the investigation, the data and conclusions of this article are considered unreliable.

The authors have been informed of this retraction but did not provide a response.
